# Delayed two steps PRP injection strategy for the improvement of fat graft survival with superior angiogenesis

**DOI:** 10.1038/s41598-020-61891-6

**Published:** 2020-03-23

**Authors:** Yuan Li, Shan Mou, Peng Xiao, Guining Li, Jialun Li, Jing Tong, Jiecong Wang, Jie Yang, Jiaming Sun, Zhenxing Wang

**Affiliations:** 1 Department of Plastic Surgery, Union Hospital, Tongji Medical College, Huazhong University of Science and Technology, 1277 Jiefang Avenue, Wuhan, 430022 China; 2 Department of Plastic Surgery, Wuhan Third Hospital (Tongren Hospital of WuHan University), Wuhan, 430060 China; 3 Wuhan Clinical Research, Center for Superficial Organ Reconstruction, Wuhan, 430022 China; 4 Department of Transfusion, Union Hospital, Tongji Medical College, Huazhong University of Science and Technology, 1277 Jiefang Avenue, Wuhan, 430022 China

**Keywords:** Preclinical research, Stem-cell research, Translational research

## Abstract

Platelet-rich plasma (PRP) has been widely used to improve the fat retention rate in autologous fat transplantation since it possesses a good angiogenesis capability *in vivo*. However, due to the short half-life of growth factors released from PRP and its uneven distribution in injected fat tissue, the strategy of PRP in fat transplantation needs further improvement. Since the capillaries started to grow into fat grafts in 1 week and vascular growth peaks in the second week after transplantation, we hypothesized that delayed two-steps PRP injection into the interior of grafts, accompanied with the extent of neovascularization might theoretically promote microvessel growth inside transplanted adipose tissue. 24 nude mice were divided into three groups: Blank group (0.35 mL fat mixed with 0.15 mL saline, N = 8), Single step group (0.35 mL fat mixed with 0.15 mLPRP, N = 8), and Two steps group (0.35 mL fat (day 0) + 0.075 mL PRP (day 7) + 0.075 mL PRP (day 14), N = 8). At 6 and 14 weeks post-transplantation, grafts were dissected, weighted, and assessed for histology, angiogenesis, fat regeneration and inflammation level. The weight and volume of the fat samples revealed no statistical difference among the three groups at 6 weeks after fat transplantation. The weight and volume of the Two steps group fat samples showed significantly higher compared to that in Blank and Single step groups at 14 weeks after fat transplantation (weight: 137.25 ± 5.60 mg versus 87.5 ± 3.90 mg,106.75 ± 2.94 mg, respectively; volume: 0.13 ± 0.01 mL versus 0.08 ± 0.01 mL, 0.09 ± 0.01 mL, respectively). Histological assessments indicated that delayed two-steps PRP injection strategy helps to improve adipose tissue content and reduce the composition of fibrous connective tissue at 14 weeks after fat transplantation. At 6 weeks and 14 weeks after transplantation, CD31 immunofluorescence indicated that delayed two-steps PRP injection strategy helps to improve angiogenesis and significantly higher compared to that in Blank and Single step groups (6 weeks: 28.75 ± 4.54 versus 10.50 ± 2.06, 21.75 ± 1.85; 14 weeks: 21.75 ± 2.86 versus 9.87 ± 2.08, 11.75 ± 1.47, respectively). Preadipocyte count indicated delayed two-steps PRP injection strategy might promote fat regeneration and significantly higher compared to that in Blank and Single step groups at 14 weeks (129.75 ± 6.57 versus 13.50 ± 3.50, 17.12 ± 6.23, respectively). In this study, we demonstrated that the novel delayed two-steps PRP injection strategy remarkably enhanced the long-term fat retention rate and improved the neovascularization extent in the interior of the fat graft. Platelet-rich plasma, Delayed two-steps injection, Angiogenesis, Fat transplantation

## Introduction

Autologous fat transplantation has become popular for its well convenience and biocompatibility in the application of soft- tissue augmentation^1^. However, the fat grafting retention rate is still less than ideal^2,3^. The results of retention rate are varied (approximately 20% to 75%)^2,3^. Many studies have investigated that the adipose tissue survival mainly depends on the neovascularization at the early stage of *in vivo* transplantation^4^.

PRP assisted fat transplantation strategy has been proven to be effective to promote early vascularization in fat grafting^5^. PRP is extracted from autologous blood that contain nearly 7.9 times the concentration of platelets than whole blood^5^. During the activation of PRP, growth factors (GF) such as TGF-β1, VEGF and FGF could be released, promoting angiogenesis, cell proliferation and differentiation^6^. Nevertheless, recent research found that the duration period for PRP only lasts for approximately 8 days after implantation, which is too short for fat survival *in vivo*^7^.

Due to early ischemia and hypoxia, adipose tissue with a diameter larger than 1200 um tend to necrosis at the central region after transplantation into the body^8^. Studies showed that implanted fat tissue can be classified into three zones from the periphery to the center at 4 weeks after transplantation: survival, regenerated, and necrotic zones^9^. The capillaries begin to grow into the survival zone at one week and peaks in the fourth week after transplantation, which is accompanied by the regeneration of adipocytes. Therefore, in order to accelerate the regeneration process, it is important to continuously induce vessel growth into the necrosis zone from the survival zone.

Based on the above literature review, we hypothesized that if we delayed injection of PRP into fat tissue at various time points, we might be able to prolong the half life of released growth factors, thus improving the angiogenesis and fat survival rates. In this study, the same quantity of activated PRP (two steps group) was injected into fat tissue of nude mice models weeks 1 and 2 after transplantation. 3 months later, specimens of the Two steps group were analyzed with histology, immunehistochemistry and immunofluorescence to demonstrate its advantages.

## Materials and Methods

### Fat tissue preparation

Fat tissue was harvested using suction-assisted lipectomy during an elective abdominoplasty performed under general anesthesia from one female patient (BMI ranging from 23–27)^5,10–13^. The donors ranged in age between 20 and 40 years with no history of disease. Written informed consent was provided by each participant and the protocol was approved by the Ethics Committee of Huazhong University of Science and Technology (approval project code: S069). All methods were performed in accordance with the relevant guidelines and regulations. To obtain lipoaspirate, we performed conventional manual liposuction to obtain 50 ml of lipoaspirate. First, we used a 2-mm cannula for tumescent infiltration (1000 mL 0.9% NaCl, 20 mL 1% lidocaine, 1 mL [1:1000] epinephrine) in the subcutaneous fat of donor. Then, we used a 3.8-mm cannula to aspirate fat particles under −0.5 bar (1 bar = 100 kPa) negative pressure. After harvested fat was washed and filtered by sterile gauze, the adipose tissue were collected.

### Preparation of PRP

PRP was prepared using whole blood collected from consented donor via two steps centrifugation method. The donors ranged in age between 20 and 40 years. Written informed consent was provided by each participant and the protocol was approved by the Ethics Committee of Huazhong University of Science and Technology (approval project code: S069). All methods were performed in accordance with the relevant guidelines and regulations. Briefly, 40 mL of blood was obtained from one patient at Wuhan Union Hospital. Then the blood was drawn into 410 ml tubes. Whole blood collected in 10 ml vacutainer containing 3.2% sodium citrate was first centrifuged at 100 g for 15 min to separate the PRP from the red and white cells, followed by second centrifugation at 600 g for 5 min to concentrate it by discarding 2 ml of plasma on top. PRP collected at the bottom of the tube was resuspended in remaining plasma stored at 4 °C for usage.

### Animal model and surgical procedures

All experiments involving animals were performed in accordance with the guidelines of the Ethics Committee of Huazhong University of Science and Technology. All animal experiments were approved by the Animal Care and Use Committee of HUST, and were performed in compliance with the National Institutes of Health Guide for the Care and Use of Laboratory Animals. 24 male BALB/c-nu nude mice (6 weeks old, 16–18 g, Beijing Vital River Laboratory Animal Technology) were randomly divided into three groups (n = 8 mice per group). Subsequently, the mice were anesthetized by means of inhaled 3% isoflurane. Lipoaspirates were injected subcutaneously at two paravertebral points of each BALB/C nude mouse: Blank group (0.35 mL fat mixed with 0.15 mL saline); Single step group (0.35 mL fat mixed with 0.15 mL PRP); Two steps group (0.35 mL fat). 7 days after transplantation, 0.075 mL PRP was injected into the interior of the fat tissue in the two steps group. Then on 14th day time point, the same injection procedure was performed on two steps group again.

**Figure 1:**
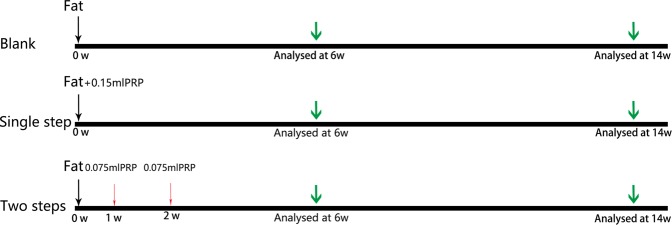
Experimental design of the two-steps injection strategy *in vivo*. 24 animals were divided into three groups. Lipoaspirates were injected subcutaneously at two paravertebral points of each BALB/C nude mouse (Blank group: 0.35 mL fat only; Single step group: 0.35 mL fat mixed with 0.15 mL PRP; Two steps group 0.35 mL fat with 0.075 mL delayed injected PRP at 1w and 2w respectively). At 6 and 14 weeks post-transplantation, grafts were dissected, weighted, and assessed for histology, angiogenesis, fat regeneration and inflammation level. Statistical analysis was performed using one-way analysis of Variance by GraphPad Prism 6.02 (GraphPad Software, Inc). P < 0.05 was chosen as the critical level of statistical significance. All data were presented as means ± standard deviation.

Briefly, fat was injected using an 18-gauge needle, the PRP was injected using a 32-gauge needle and the mini-incisions were closed with 6-0 nylon sutures. At scheduled times (6 and 14 weeks), four randomly selected animals (n = 2 samples per animal) in each treatment group were euthanized, and the samples were harvested for the following analyses. The experiment has repeated for three times (Fig. 1).

### Histologic evaluation

Animals (n = 8 samples per group) each group were sacrificed at 6 or 14 weeks post-transplantation. Each sample was weighed, and the volume was measured by the liquid overflow method^5,10^. Samples were fixed in 4% paraformaldehyde, dehydrated, and embedded in paraffin. Tissue blocks were sectioned, photographed using the digital camera (Nikon DS-Ri2, Japan). We used Image-Pro Plus 6.0 to calculate and identify the presence of cysts and vacuoles, the percentage area of complete adipose tissue and the percentage area of existing connective tissue per low vision (40 randomized fields for each group at 6 weeks and 14 weeks time point) (100× magnification)^12^. The evaluations were performed by two researchers in a double-blinded manner.

### Assessment of capillary density

Paraffin-embedded sections were incubated with primary antibodies, and rabbit anti–human CD31 (Abcam, Cambridge, Britain, ab28364, 1:50). Slides were observed under light microscope (Nikon Ni-U, Japan) and the capillaries were counted in 20 randomized fields on each section (200× magnification) by two researchers who were blinded to the study groups.

### Immunohistochemistry

Tissue sections were immunofluorescently stained with the following primary antibodies: rat anti-mouse Mac2 (1:200; Cedarlane Corp., Burlington, Ontario, Canada), rabbit anti-mouse CD206 (1:300; Abcam, Cambridge, MA, USA), and guinea pig anti-mouse perilipin (1:200; Progen, Heidelberg Germany), After washing, the samples were incubated with donkey anti-rat-555 IgG (1:200; Abcam) and goat anti-rabbit-430 IgG (1:200; Invitrogen, North Ryde, NSW, Australia). Nuclei were stained with DAPI 33342 (Solarbio, D8200, Beijing),and then samples were examined under a light microscope (Nikon Ni-U, Japan). To assess the regeneration of adipose tissue, the preadipocytes^13^ were counted in 20 randomized fields on each section (200× magnification). To investigate the effect of two injection strategies on the extent of adipose tissue macrophage infiltration, we evaluated the fluorescence staining of M1(red) M2(yellow) macrophages at 100x magnification. A blinded method was adopted in our experiment. The sample for each mark was unknown by the investigator.

### Statistical analysis

All data were presented as means ± standard deviation. Statistical analysis was performed using one-way analysis of Variance by GraphPad Prism 6.02 (GraphPad Software, Inc, San Diego, Calif) and used post-hoc correction for pairwise comparisons. P < 0.05 was chosen as the critical level of statistical significance.

## Results

### Fat tissue survival assessment

**Figure 2:**
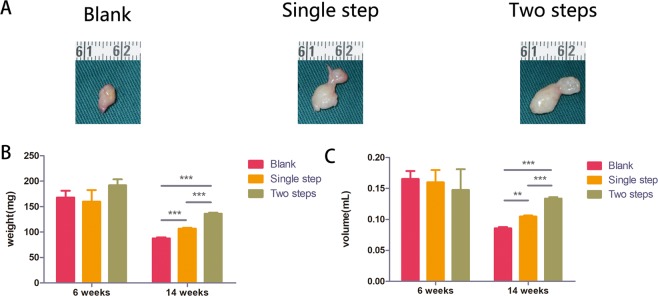
Gross view and retention rate comparisons of fat grafts after 14 weeks transplantation. Representative samples in the three groups are shown (**A**). The Two steps group (**A**. right) showed better tissue integrity than the other two groups. Samples weight and volume were analyzed at 6w and 14w after fat transplantation (**B,C**). There was no statistical difference in grafts weight between the three groups at 6 weeks (p > 0.05) (**B**). At 14 weeks, the survival weights of the Two steps group were significantly greater than those of the Single step and the Blank group (***p < 0.001, scale bar = 1 cm) (**B**). There was no statistical difference in grafts volume between the three groups at 6 weeks (p > 0.05) (**C**). At 14 weeks, the survival volume of the Two steps group were significantly greater than those of the Single step and the Blank group (***p < 0.001, scale bar = 1 cm) (**C**).

Poor surface vascularization and visible oil cysts were found in the Blank group. The Single step group revealed more visible oil cysts at 14 weeks (Fig. 2A). There was no difference in appearance between the three groups of fat samples at 6 weeks (Supplemental Fig. 1). The mean values and standard deviations for weight maintenance at the time of 6 weeks and 14 weeks after transplantation for the Blank group, the Single step group and the Two steps group were 164.62 ± 15.26 mg, 163.83 ± 28.39 mg, 191.88 ± 20.31 mg (6 weeks) and 87.5 ± 3.90 mg, 106.75 ± 2.94 mg, 137.25 ± 5.60 mg (14 weeks) (Fig. 2B). There was no statistical difference in grafts weight between the three groups at 6 weeks (p > 0.05). At 14 weeks, the weight of the Two steps group were significantly greater than those of the Single step and the Blank group (p < 0.05) (Fig. 2B). The volume of transplanted samples in the Blank group, the Single step group and the Two Steps group at 6 weeks and 14 weeks after fat transplantation were 0.16 ± 0.02 mL, 0.16 ± 0.02 mL, 0.14 ± 0.05 mL (6 weeks) and 0.08 ± 0.01 mL, 0.09 ± 0.01 mL, 0.13 ± 0.01 mL (14 weeks) (Fig. 2C). There was no statistical difference in grafts volume between the three groups at 6 weeks (p > 0.05) (Fig. 2C). At 14 weeks, the volume of the Two steps group were significantly greater than those of the Single step and the Blank group (p < 0.05) (Fig. 2C).

### Histologic evaluation

**Figure 3:**
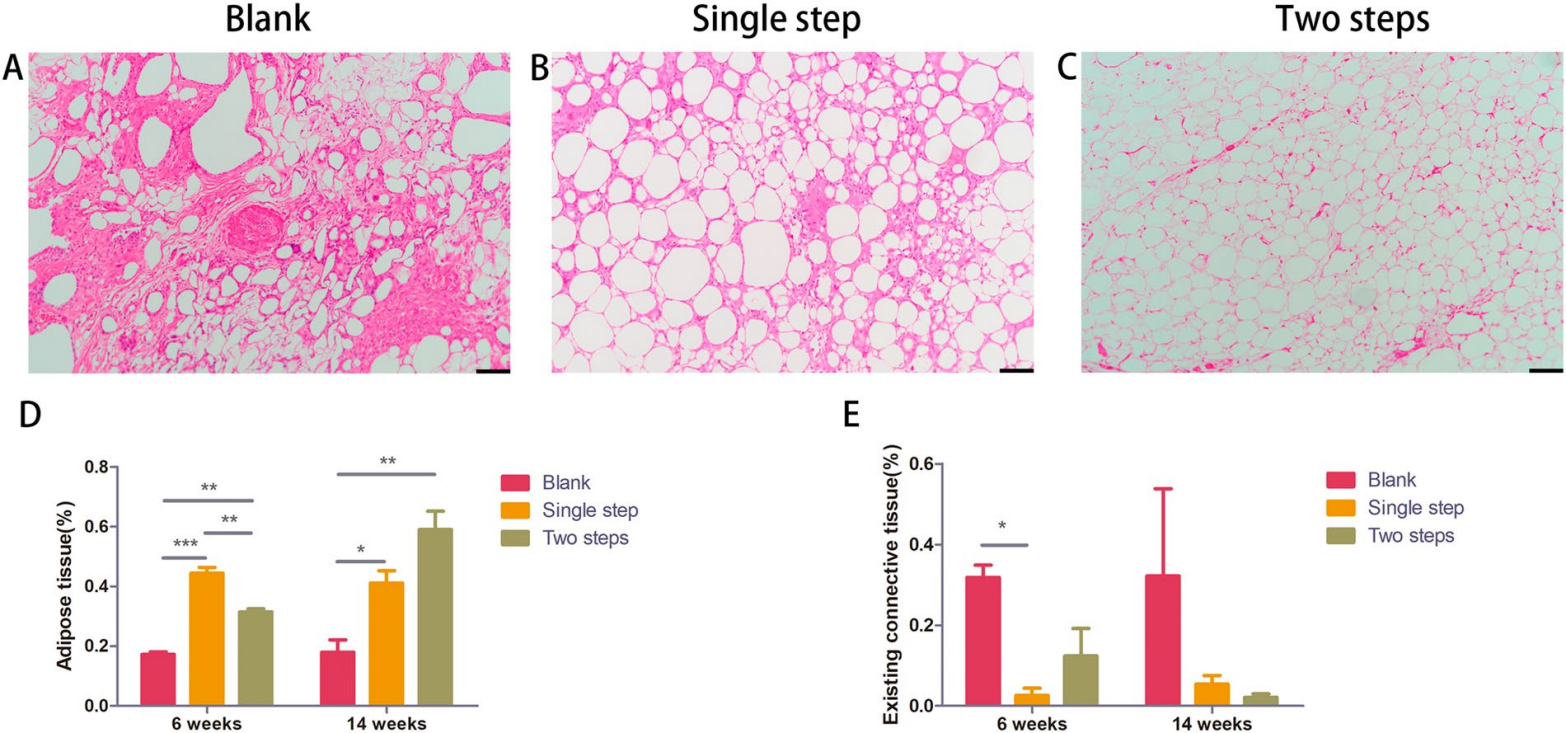
HE staining of grafted fat tissue at 14 weeks (**A–C**). The Two steps group (**C**) showed a normal adipose structure with hardly any necrosis or fibrosis. Morever, mature adipocytes were uniform in size. Histologic characteristics were evaluated 6 weeks and 14 weeks after transplantation (Fig. 3D,E). At 6 weeks, there were more adipose tissue in the Single group than other two groups (**p < 0.01, scale bar = 100 μm) (Fig. 3D) and the percentage area **o**f existing connective tissue was the least in the Single step group among all three groups (*p < 0.05, scale bar = 100 μm) (Fig. 3E). At the 14 weeks time point, the amount of adipose tissue in the Two steps group was significantly higher than the other two groups (**D**) (**P < 0.01, scale bar = 100 μm), while the amount of observed connective tissue was the lowest (**E**).

Hematoxylin and eosin staining was used to observe the morphologic difference of fresh lipoaspirates among the three groups. At 6 weeks, Single step group showed best morphological integrity among all three groups (Supplemental Fig. 2). At 14 weeks, the fat grafts in the Two steps group exhibited the best morphological integrity among all three groups (Fig. 3A–C). Histologic characteristics were evaluated 6 weeks and 14 weeks after transplantation (Fig. 3D,E). The percentage of adipose tissue per optical field was calculated at the time of 6 weeks and 14 weeks after transplantation for the Blank group, the Single step group and the Two steps group were 18% ± 3%, 43% ± 6%, 34% ± 2% (6 weeks) and 22% ± 6%, 35% ± 10%, 45% ± 6% (14 weeks) (Fig. 3D). At 6 weeks, there were more adipose tissue in the Single group than other two groups (p < 0.05) (Fig. 3D). The percentage was the highest in the Two steps group among all three groups at 14 weeks (Fig. 3D). The percentage area of existing connective tissue per optical field was calculated at the time of 6 weeks and 14 weeks after transplantation for the Blank group, the Single step group and the Two steps group were 30.4% ± 16.7%, 3.3% ± 1.1%, 21.4% ± 13.5% (6 weeks) and 33% ± 26.7%, 5.4% ± 3.0%, 1.3% ± 0.2% (14 weeks) (Fig. 3E). At 6 weeks, the percentage area of existing connective tissue was the least in the Single step group among all three groups (Fig. 3E). The percentage area of existing connective tissue was the least in the Two steps group among all three groups at 14 weeks (Fig. 3E).

### Viability of adipocytes in transplanted fat tissue

**Figure 4:**
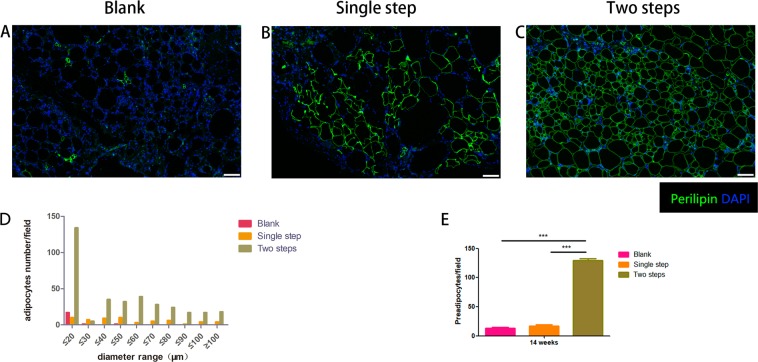
Perilipin staining of fat grafts at 14 weeks (**A–C**). Perilipin-positive adipocytes were relatively small-sized in the Two steps group compared to the other two groups and the preadipocytes varied in different sizes. Quantitative measurements of adipocytes number in the central part of the specimens showed that the Two steps group had the most preadipocytes (<20 μm) (**D**). Preadipocytes number of the Two steps group were significantly greater than those of the Single step and the Blank group (***p < 0.001) (**E**). Scale bar = 100 μm.

Samples were stained for perilipin (cytoplasm of viable adipocytes, green) and DAPI (cell nuclei, blue) to assess the viability of the adipocytes in the grafts at 6 weeks and 14 weeks after transplantation. At 6 weeks, Single step group showed the most green-stained area (Supplemental Fig. 3). The Two steps group exhibited more viable adipocytes than the Blank group and the Single step group at 14 weeks (Fig. 4A–C). To evaluate the regeneration of adipose tissue, we measured the diameters of adipocytes in different diameters at 14 weeks (Fig. 4D). The mean values and standard deviations for preadipocytes (diameter <20 μm) number at the time of 14 weeks after transplantation for the Blank group, the Single step group and the Two steps group were: 13.50 ± 3.50, 17.12 ± 6.23, 129.75 ± 6.57 (Fig. 4E). Preadipocytes number of the Two steps group were significantly greater than those of the Single step and the Blank group (p < 0.05) (Fig. 4E).

### Capillary density assessment in grafted fat tissue

**Figure 5:**
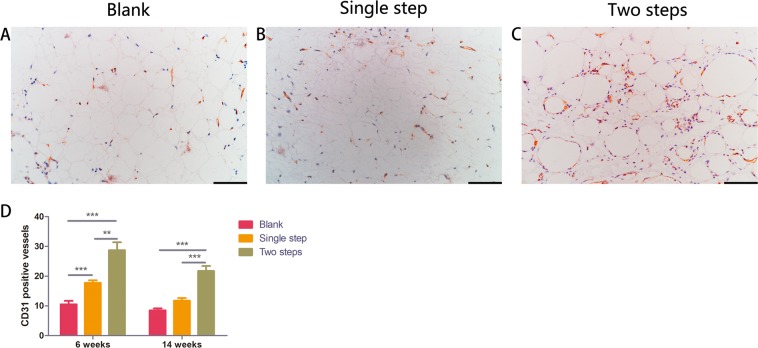
CD31 immunohistology staining of fat grafts 14 weeks (**A–C**). Quantitative statistics results confirm that CD31 positive cells are much higher in the Two steps group than the other two groups at 6 weeks and 14 weeks (**D**). (**p < 0.01; ***p < 0.001, respectively) Scale bar = 100 μm.

Immunohistochemical staining of CD31 revealed the presence of neoangiogenesis. The red stained tubular structure is the blood vessel (Fig. 5A–C). The mean values and standard deviations for vessel number at the time of 6 weeks and 14 weeks after transplantation for the Blank group, the Single step group and the Two steps group were: 10.50 ± 2.06, 21.75 ± 1.85, 28.75 ± 4.54 (6 weeks) and 9.87 ± 2.08, 11.75 ± 1.47, 21.75 ± 2.86 (14 weeks) (Fig. 5D). Vessel number of the Two steps group were significantly greater than those of the Single step and the Blank group at 6 weeks and 14 weeks (p < 0.05) (Fig. 5D).

### Macrophage infiltration in the grafts

**Figure 6:**
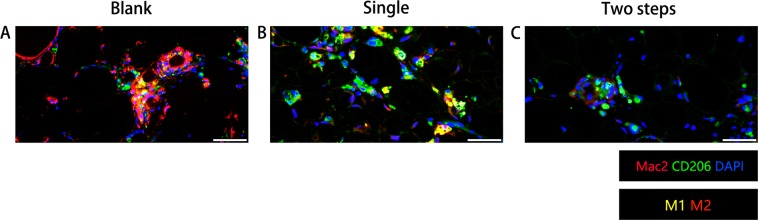
Histological analysis of macrophages in specimens at 14 weeks. M1 macrophages were characterized by positive Mac2 staining (red) and negative CD206 staining (green), while M2 macrophages were found with both positive staining in Mac2 and CD206 (yellow). In the Blank group (**A**), dead adipocytes and oil drops were surrounded by macrophages (M1, M2) in a typical “crown-like” structure. Meanwhile few positive M1 or M2 macrophages were found in the Two steps group (**C**). There were fewer M1 (red) and M2 (yellow) macrophages in the Two steps group than in the Single step group. Immunofluorescence staining of Mac2 + M1 (red), Mac2+/CD206+ M2(yellow) which showed that macrophages were successfully activated in Single step group and depleted in Two steps group at week 14. Scale bar = 100 μm.

Macrophages were visualized by immunohistochemistry staining for Mac2 (red) and CD206 (green) (Fig. 6). At 14 weeks after grafting, in the Blank group oil droplets with the size of adipocytes that were not yet completely absorbed were surrounded by a multilayer of infiltrating macrophages (M2 (Mac2+/CD206+, yellow), M1 (Mac2, red)), these macrophages clustered around large oil droplets to form a typical “crown-like” structure (Fig. 6A). Meanwhile, some oil droplets were completely absorbed, leaving only a multilayer of M2 (yellow) macrophages in the Two steps group (Fig. 6C). The level of inflammatory infiltration in the Single step group was higher than the Two steps group but less than the Blank group (Fig. 6B). At 6 weeks, the obvious red staining (M1) of the Blank group was observed, and the degree of inflammatory infiltration was much higher than that of the Single step group and the Two step group. It was also found that the Single step group was dominated by yellow staining (M2), the two steps group was dominated by red staining (M1) (Supplemental Fig. 5).

## Discussion

In order to improve the utilization rate of PRP, we improved the strategy of using PRP according to the time of transplantation of fat vascularization. This experiment is designed into three groups: Blank group, Single step group and Two steps group (Fig. 1). The volume retention rate of fat samples at 6 weeks and 14 weeks after fat transplantation showed that delayed two steps PRP injection strategy can improve the transplantation of fat volume retention rate. At 14 weeks, the weight of the Two steps group were significantly greater than those of the Single step and the Blank group (p < 0.05) (Fig. 2B). At 14 weeks, the volume of the Two steps group were significantly greater than those of the Single step and the Blank group (p < 0.05) (Fig. 2C). PRP could create a first-rank micro-environment that promoted cell growth and cell differentiation. Thus it was considered as an useful mean for fat transplantation^14^. However, recent studies reported that breast fat grafting produced no better impact when the combination of PRP with fat grafts than fat grafting alone^15,16^. This may be attributed to the insufficient vascular ingrowth in the center of fat tissue^16–18^. Understanding how to revascularize in the central region of adipose tissue is essential for long-term survival in a large volume of fat tissue transplantion. Transplantation of adipose tissue vascular growth begins 1 week after transplantation and peaks at 4 weeks, so we hypothesized that supplementation with PRP within the time of graft growth may increase PRP utilization. The experimental results confirm our hypothesis.

To further judge the effect of delayed two steps PRP injection strategy on the integrity of adipose tissue in fat samples, we analyzed the results of HE staining of adipose tissue. Single step group showed best morphological integrity among all three groups (Supplemental Fig. 2) and there were more adipose tissue in the Single group than other two groups (p < 0.05) (Fig. 3D) at 6 weeks. The percentage was the highest in the Two steps group among all three groups at 14 weeks (Fig. 3D). We speculated that delayed two steps PRP injection strategy was more effective in improving the integrity of transplanted adipose tissue than the traditional mixed PRP injection strategy. However, the first 3 months after fat transplantation is a dynamic process^17^. According to previous reports, grafted fat could be divided into three regions, from the periphery to the center: (1) survival region (superficial); (2) regeneration region (middle); and (3) necrosis region^17^. If we inject PRP into the adipose tissue on the seventh day after fat transplantation to interfere with the dynamic process of adipose tissue after transplantation, it should be possible to reduce the fibrous necrosis area to a certain extent, and increase the adipose tissue survival area and regeneration area. The statistical results of adipose tissue content confirm this to some extent.

Delayed two steps PRP injection strategy may promote the formation of preadipocytes in transplanted adipose tissue to some extent. Statistical analysis of the number of preadipocytes showed that preadipocytes number of the Two steps group were significantly greater than those of the Single step and the Blank group (p < 0.05) (Fig. 4E). Delayed two steps PRP injection strategy promotes the regeneration of adipose tissue to a certain extent. Surviving adipose tissue was located superficially below the tissue margin to a depth of 100 to 300 μm^18^. In the regeneration zone, new small preadipocytes (<20 μm) appear around dead adipocytes within 1 to 2 weeks post transplantation^19^. Fat graft angiogenesis and regeneration begin 1 week after fat transplantation and, peaks at 4 weeks^20,21^. The Two steps group was injected with PRP into the interior of the grafts on the first week (capillaries and preadipocytes begin to regenerate) and second week (the growth rate of capillaries and preadipocytes gradually reached the peak after transplantation). For perilipin staining, the green positive area of the grafts obtained by the Two steps group was the highest among the three groups (Fig. 4). At the same time, the number of preadipocytes was also the highest among the three groups. It could partly speculated that the delayed two-steps PRP injection strategy improves the final volume retention rate by promoting regeneration of the transplanted fat regeneration zone (Fig. 2B). The reason was most likely because the injection strategy could be replenish growth factors in the process of vessels growth, so that the growth factor can be correctly utilized. As is reported that the innermost zone was the necrotizing area, where new adipocytes were rare. We could partly speculated that the regeneration of adipocytes is also partly promoted by the internal vascularization of adipose tissue.

In order to evaluate the effect of Delayed two steps PRP injection strategy on the degree of internal vascularization of adipose tissue, statistical analysis was performed on the number of blood vessels in CD31 stained sections. Vessel number of the Two steps group were significantly greater than those of the Single step and the Blank group at 6 weeks and 14 weeks (p < 0.05) (Fig. 5D). Delayed two steps PRP injection strategy may be due to the replenishment of growth factors necessary for blood vessel growth in adipose tissue in accordance with the time of blood vessel growth in adipose tissue, making this strategy more effective in promoting the growth of blood vessels in transplanted adipose tissue than the traditional strategy of mixed PRP transplantation. It might because the injection strategy partly promoted early angiogenesis of transplanted adipose tissue in the interior area, which contributed to interior fat regeneration and increase the final volume retention rate.

Macrophages have been shown to be one of an inflammatory cell that predominantly exists in the transplanted micro-environment^21,22^. By week 1, most of the macrophages were M1 polarized, indicating a “inflammatory phase” during which dead cells were cleared. From week 2 to week 4 and after transplantation, M2 macrophages slowly became dominant, indicating the beginning of the “regeneration phase”^22^. However, the regeneration of fat sometimes halted 8 weeks after transplantation, for the reason that large oil droplets formed by necrotic adipocytes hampered cell proliferations^21,22^. Our results showed that, 14 weeks after transplantation, more M2 macrophages gathered around the oil droplets in the Two steps group (Fig. 6C). As is reported that M2 macrophages could mitigate the inflammatory response and promote the regeneration. We speculated that this could partly explain the superior volume retention rate in two steps group. While the Blank group still showed obvious coronary structures, indicating that a severe inflammatory reaction had occurred (Fig. 6A). Therefore, delayed two-steps PRP injection strategy might be more effective in alleviating inflammatory responses.

Last but not least, we have demonstrated that the Two steps injection strategy can improve the volume retention of 0.35 ml of transplanted fat. We are encouraged by macrophage color display, suggesting that two-step injection of PRP may promote the regeneration of transplanted fat. However, 0.35 ml fat transplantation is not enough to meet the clinical requirement for fat transplantation, and we may need to further demonstrate that this injection strategy may increase the volume of fat transplantation.

## Conclusion

In this study, we demonstrated that the delayed two-steps PRP injection strategy could remarkably enhance the long-term fat retention rate and improve neovascularization in the interior of fat grafts, compared with PRP mixed with graft fat. This novel delayed two-step PRP injection strategy provides an improved scenario for plastic surgeons in large-volume fat transplantation applications.

## Supplementary information

Supplementary Data

Supplementary Figures

## Data Availability

The datasets used and/or analyzed during the current study are available from the corresponding author on reasonable request.
