# Supplementary material for: Delayed two steps PRP injection strategy for the improvement of fat graft survival with superior angiogenesis
Source: Sci Rep. 2020 Mar 23;10:5231. doi: 10.1038/s41598-020-61891-6 (PMC7089949; doi:10.1038/s41598-020-61891-6)

# Delayed two steps PRP injection strategy for the improvement of fat graft survival with superior angiogenesis

Yuan Li[a], Shan Mou[a], Peng Xiao[a], Guining Li[b],Jialun Li[a] , Jing Tong[a], Jiecong Wang[a], Jie Yang [a,*], Jiaming Sun[a,*], Zhenxing Wang[a,*]

a. Department of Plastic Surgery, Union Hospital, Tongji Medical College, Huazhong University of Science and Technology, 1277 Jiefang Avenue, Wuhan 430022, China.

b. Department of Transfusion, Union Hospital, Tongji Medical College, Huazhong University of Science and Technology, 1277 Jiefang Avenue, Wuhan, 430022, China

***Corresponding author:**

Dr. Zhenxing Wang, MD, PhD

Department of Plastic Surgery, Union Hospital, Tongji Medical College, Huazhong University of Science and Technology, 1277 Jiefang Avenue, Wuhan 430022, China.

Phone/ Fax: +86-027-85726114; E-mail: wangzhenxing@hust.edu.cn

Dr. Jiaming Sun, MD, PhD

Department of Plastic Surgery, Union Hospital, Tongji Medical College, Huazhong University of Science and Technology, 1277 Jiefang Avenue, Wuhan 430022, China.

Phone/ Fax: +86-027-85726114; E-mail: sunjm1592@sina.com

Dr. Jie Yang, MD, PhD

Department of Plastic Surgery, Union Hospital, Tongji Medical College, Huazhong University of Science and Technology, 1277 Jiefang Avenue, Wuhan 430022, China.

Phone/ Fax: +86-027-85726114; E-mail: abrams18@163.com

## The email address of all authors :


| | |
|---|---|
| Yuan Li | 915322062@qq.com |
| Shan Mou | tjmedms@163.com |
| Peng Xiao | whpjtx@163.com |
| Guining Li | guining_119@yeah.net |
| Jialun Li | jl.licn@foxmail.com |
| Jing Tong | 1003219280@qq.com |
| Jiecong Wang | wangjiecong1982@sina.com |
| Jie Yang | abrams18@163.com |
| Jiaming Sun | sunjm1592@sina.com |
| Zhenxing Wang | benjamin.wzx@163.com |


Supplemental figure 1

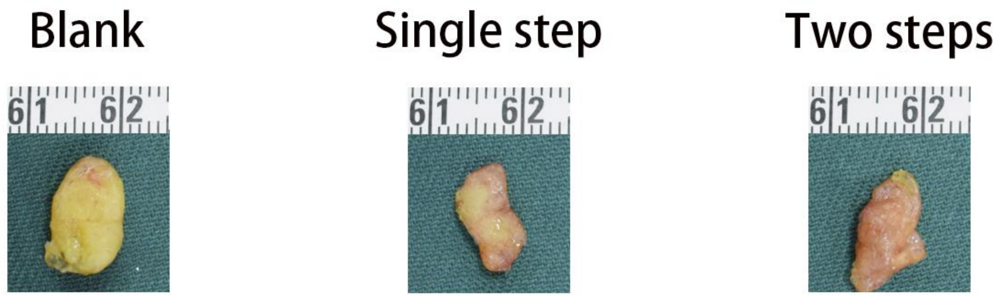

Supplemental figure 2

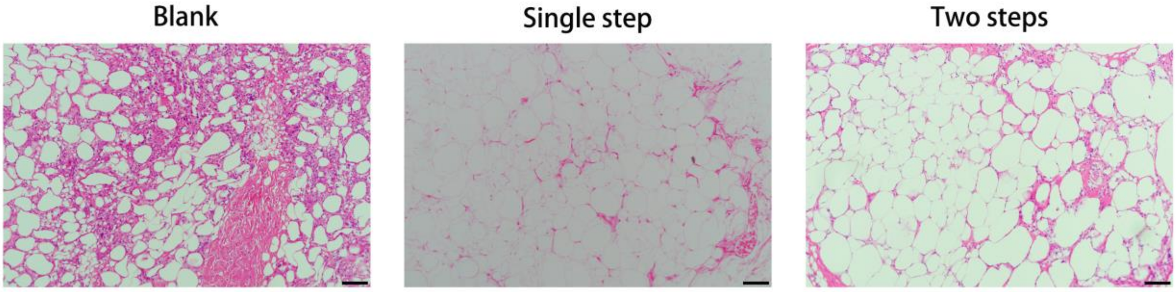


Supplemental figure 3

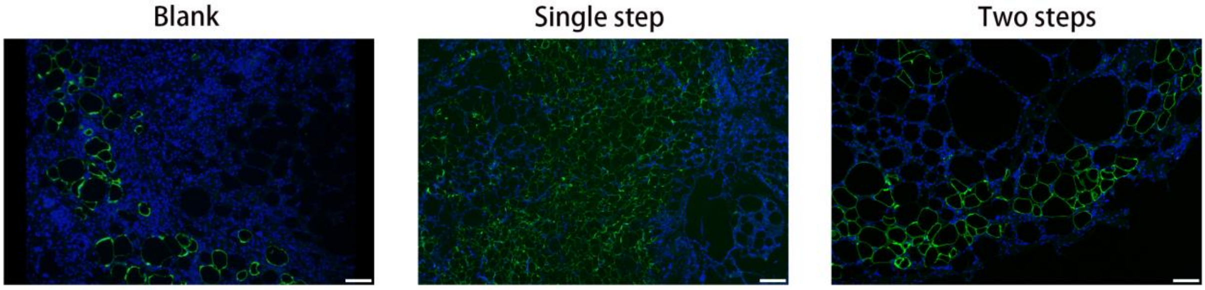


Supplemental figure 4

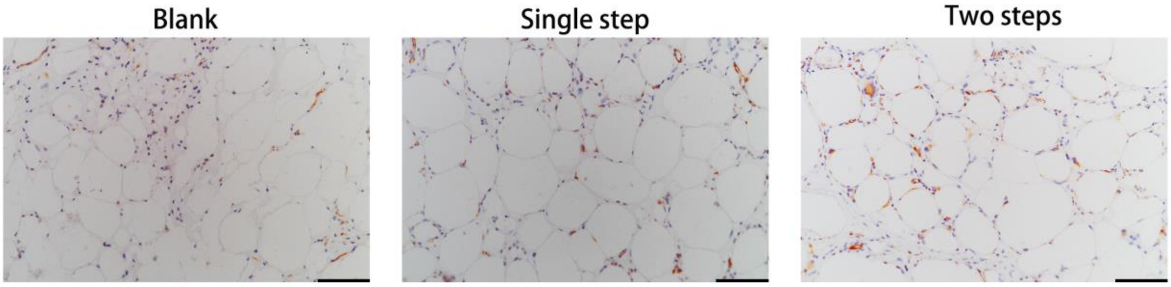

Supplemental figure 5

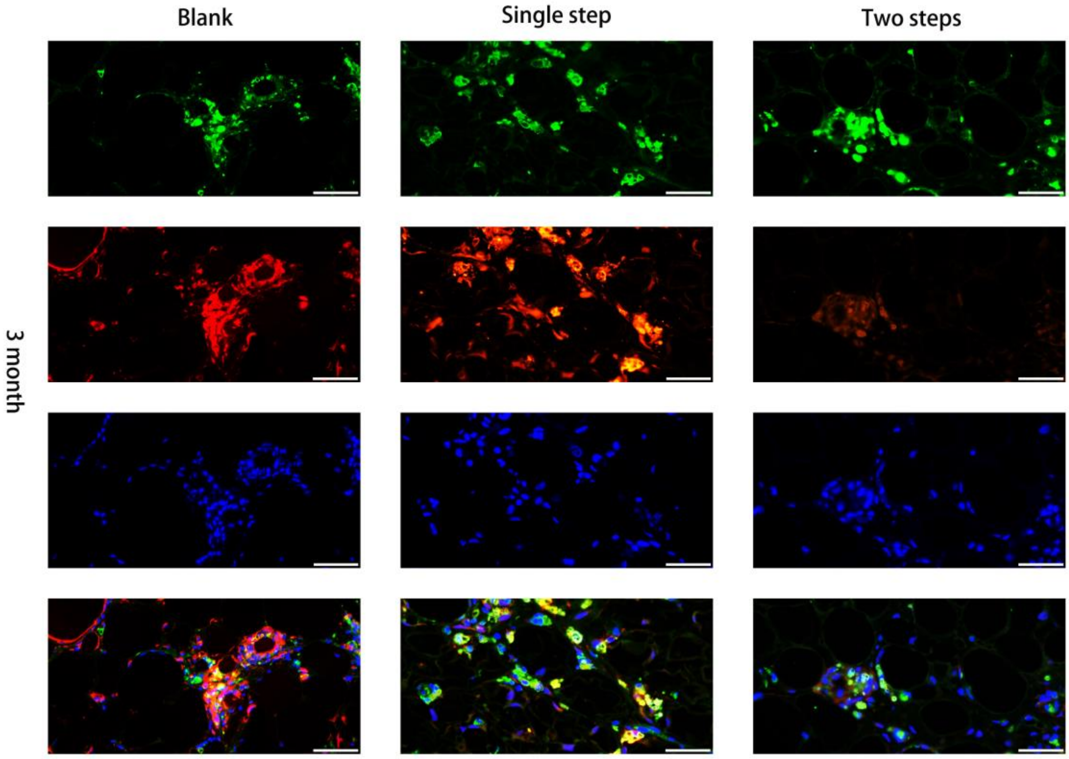


Supplemental figure 6

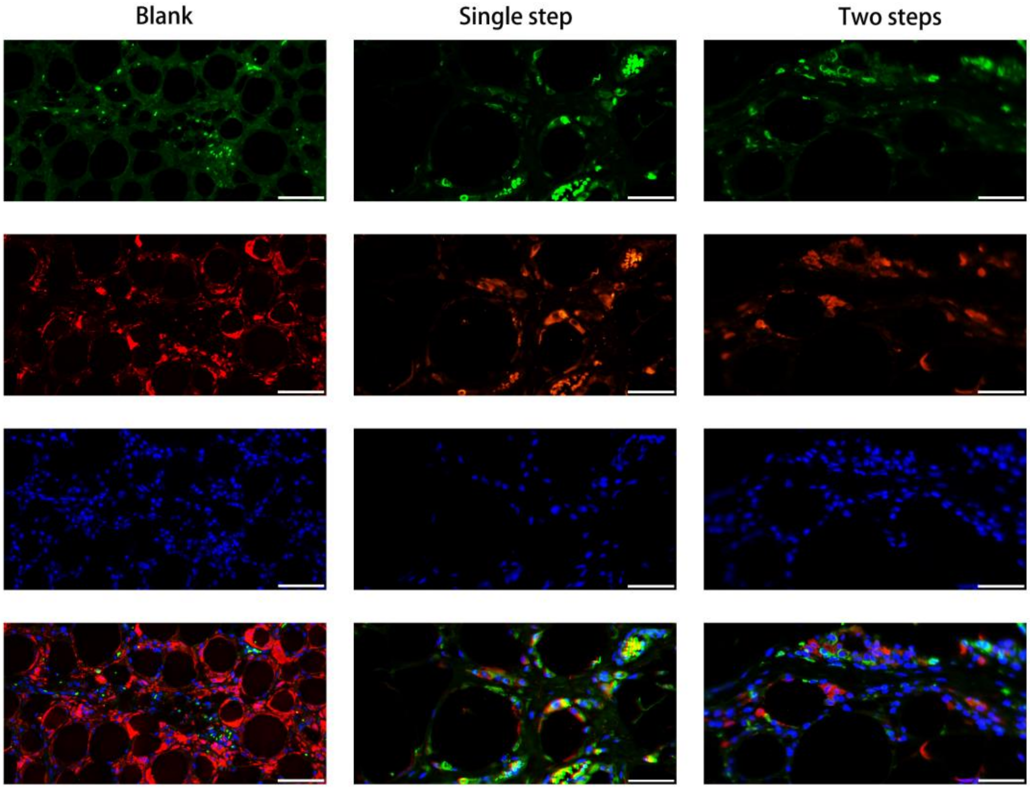

Supplement: Supplementary file 2 — Supplementary Figures [file 41598_2020_61891_MOESM2_ESM.pdf]
